# ReishiMax, mushroom based dietary supplement, inhibits adipocyte differentiation, stimulates glucose uptake and activates AMPK

**DOI:** 10.1186/1472-6882-11-74

**Published:** 2011-09-19

**Authors:** Anita Thyagarajan-Sahu, Brandon Lane, Daniel Sliva

**Affiliations:** 1 Cancer Research Laboratory, Methodist Research Institute, Indiana University Health, 1800 N Capitol Ave, E504, Indianapolis, IN 46202, USA; 2 Department of Medicine, School of Medicine, Indiana University, Indianapolis, IN, USA; 3 Indiana University Simon Cancer Center, Indianapolis, IN, 46202, USA

## Abstract

**Background:**

Obesity is a health hazard which is closely associated with various complications including insulin resistance, hypertension, dyslipidemia, atherosclerosis, type 2 diabetes and cancer. In spite of numerous preclinical and clinical interventions, the prevalence of obesity and its related disorders are on the rise demanding an urgent need for exploring novel therapeutic agents that can regulate adipogenesis. In the present study, we evaluated whether a dietary supplement ReishiMax (RM), containing triterpenes and polysaccharides extracted from medicinal mushroom *Ganoderma lucidum*, affects adipocyte differentiation and glucose uptake in 3T3-L1 cells.

**Methods:**

3T3-L1 pre-adipocytes were differentiated into adipocytes and treated with RM (0-300 μg/ml). Adipocyte differentiation/lipid uptake was evaluated by oil red O staining and triglyceride and glycerol concentrations were determined. Gene expression was evaluated by semi-quantitative RT-PCR and Western blot analysis. Glucose uptake was determined with [^3^H]-glucose.

**Results:**

RM inhibited adipocyte differentiation through the suppresion of expression of adipogenic transcription factors peroxisome proliferator-activated receptor-γ (PPAR-γ), sterol regulatory element binding element protein-1c (SREBP-1c) and CCAAT/enhancer binding protein-α (C/EBP-α). RM also suppressed expression of enzymes and proteins responsible for lipid synthesis, transport and storage: fatty acid synthase (FAS), acyl-CoA synthetase-1 (ACS1), fatty acid binding protein-4 (FABP4), fatty acid transport protein-1 (FATP1) and perilipin. RM induced AMP-activated protein kinase (AMPK) and increased glucose uptake by adipocytes.

**Conclusion:**

Our study suggests that RM can control adipocyte differentiation and glucose uptake. The health benefits of ReishiMax warrant further clinical studies.

## Background

Obesity is increasing at an alarming rate in the developed as well as developing countries [1]. In the United States obesity is increasing not only in adults but also in children [2]. Among various factors which contribute to the likelihood of death, obesity has escalated the chances of death by 20% and it has lately surpassed smoking as the number one cause of death in the US [3,4]. According to survey carried out in 2007-2008 by the National Health and Nutrition Examination Survey (NHANES) nearly 32.2% of adult men and 35.5% of adult women were obese [5]. There are numerous epidemiological studies indicating that obese humans are at elevated risk of developing non-insulin-dependent (type 2) diabetes mellitus, hypertension, cancer and atherosclerosis [3,6,7].

There are several lines of evidence to show that obesity triggers the dysregulation of the endocrine function of the adipose tissue [8,9]. This suggests that adipose tissue is no longer considered to be an inert tissue solely responsible for energy store, instead is recognized as a major secretory organ, releasing a variety of adipocytokines such as adiponectin, leptin, resitin and visfatin [10]. These adipocytokines provide the link between obesity, insulin resistance and inflammatory disorders [10,11]. Differentiation of fibroblasts into adipose tissue requires chronological changes in the expression of numerous genes [12-14]. The initial events are orchestrated by several transcription factors, CCAAT/enhancer binding protein-α (C/EBP-α), sterol regulatory element binding protein-1c (SREBP-1c) and peroxisome proliferators-activator receptor-γ (PPAR-γ). PPAR-γ is involved in the regulation of genes controlling lipid uptake and is also a "master" regulator that triggers the complete process of adipogenesis [15]. These proteins participate in a transcriptional cascade that controls the expression of a number of genes which are essential in the lipid accumulation in adipocytes during the process of differentiation [16,17]. Sterol regulatory element binding proteins (SREBPs) are transcription factors that have been associated with lipogenesis regulating the expression of acetyl-CoA carboxylase (ACC), fatty acid synthase (FAS) and acyl-CoA synthase (ACS) [18]. The expression of the above factors alone cannot promote differentiation of pre-adipocytes, but when co-expressed with on adipocytes expressing PPAR-γ cell differentiation is enhanced [19-22]. Differentiated adipocytes also secrete cytokines tumor necrosis factor-α (TNF-α), and interleukins (IL-6 and IL-1β) which are major regulators of adipocyte metabolism [23]. Hence, adipose tissue plays an important role in homeostasis of energy and is emerging as a major drug target for obesity and obesity mediated metabolic disorders [24-26]. Recent studies have shown a strong link between obesity and type 2 diabetes and insulin resistance [27]. Therefore, various pharmacologic agents (e.g. thiazolidinediones and metformin) are used to enhance insulin sensitivity [28]. A main mode of action of a common type 2 diabetes drug, metformin is an activation of AMP-activated protein kinase (AMPK) in skeletal muscles and hepatocytes [29].

Medicinal mushroom *Ganoderma lucidum*, which is well recognized by the Traditional Chinese Medicine (TCM) [30-33], is globally used as a popular dietary supplement. We have previously demonstrated that ReishiMax (RM), a dietary supplement containing triterpenes and polysaccharides extracted from medicinal mushroom *G. lucidum*, is effective in inhibiting the proliferation, invasive behavior and angiogenesis in different cancer models [34-39]. In the present study we evaluated the effect of RM on the adipocyte differentiation and glucose uptake in adipocytes. Our data suggest that RM can be used as a natural agent to control obesity and associated type 2 diabetes.

## Methods

### Materials

3T3-L1 preadipocytes, Dulbecco's modified Eagle's (DMEM) medium, Trypsin-EDTA were obtained from American Type Culture Collection (ATCC, Manassas, VA, USA). Fetal calf serum (FCS) was purchased from Colorado serum company (Denver, CO, USA). Fetal bovine serum (FBS), Penicillin and streptomycin were obtained from Invitrogen (Carlsbad, CA, USA). Insulin (INS), dexamethasone (DEX), isobutyl-methylxanthine (IBMX), oil red O, isopropanol and dimethyl sulphoxide (DMSO) were received from Sigma (St. Louis, MO, USA). Dulbecco's Phosphate Buffered Saline (DPBS) was obtained from Lonza (Walkerville, MD, USA). ReishiMax (RM) was supplied by Pharmanex (Provo, UT). RM is a standardized *Ganoderma lucidum *extract containing 6% triterpenes and 13.5% polysaccharides and the extraction procedure was previously described [40]. RM stock solution was prepared in DMSO.

### Cell culture and adipocyte differentiation

3T3-L1 preadipocytes were maintained in DMEM, supplemented with 10% FCS, penicillin (100 units/ml) and streptomycin (100 μg/ml) at 37°C in humidified atmosphere with 5% CO_2_. Cell differentiation was initiated as previously described [41]. Briefly, cells were grown in 6 well plates until 2 days post-confluence. Differentiation was induced by adding 1 μg/ml of insulin, 0.25 μM of dexamethasone and 0.5 mM isobutyl-methylxanthine in DMEM with 10% FBS. The INS-DEX-IBMX medium was replaced after 2 days to medium with insulin alone for another 2 days after which they were supplemented with 10% FBS every other day thereafter. To evaluate the role of RM on lipid accumulation 3T3-L1 cells were treated with 0-300 μg/ml of RM along with the differentiation medium containing INS-DEX-IBMX. The differentiated cells were used after 9 days of initiating the differentiation. All cells, including controls cells, were treated with the same concentration of DMSO (0.3%).

### Oil red O staining

The lipids accumulated in the adipocytes were quantified after staining the 3T3-L1 cells with oil red O staining [42]. Briefly, after differentiation the cells were fixed with 10% formalin for 1 hour and washed with 60% iso-propanol. After air drying the plates were stained with oil red O working solution (6 parts of 0.35% stock Oil red O and 4 parts of distilled water) for 30 min and rinsed with water. Images were obtained using an Olympus CX40 microscope (Center Valley, PA, USA) at 20× magnification, picture frame software from Optronics (Goleta, CA, USA) was used to obtain all images at similar intensities. To quantify the amount lipid uptake, the oil red O stain was eluted by adding 100% iso-propanol for 10 min, and the absorbance measured at 510 nm on a microplate spectrophotometer using the Gen5 software by BioTek Instruments (Winooski, VT, USA).

### Analysis of triglycerides (TG)

During differentiation 3T3-L1 cells were treated with RM (0-300 μg/ml). Subsequently the cells were rinsed with PBS, scrapped and collected [43,44]. Triglycerides (TG) were quantified in the extract according to manufacture's protocol (Triglyceride GPO-Trinder Kit, St. Louis, MO, USA).

### Lipolysis determination

Lipolysis was determined as the amount of free endogenous glycerol released after RM treatment (0-300 μg/ml) into the conditioned medium. Glycerol concentration was measured using the GPO-Trinder assay kit (Sigma, St Louis, MO, USA), according manufacture's protocol.

### Isolation and analysis of RNA

Total RNAs were isolated from 3T3-L1 adipocytes using RNeasy Mini Kit (Qiagen, Valencia, CA) after the cells were treated with RM (0-300 ug/ml) during the differentiation process. Each sample of first-strand cDNA was synthesized from 2 μg total RNA using SuperScript III first-strand synthesis system (Invitrogen, Carlsbad, CA, USA) according to manufacturer's protocol using DNA thermal cycler 480 (Perkin Elmer, Emeryville, CA, USA). The cDNA was amplified using Platinum PCR master mix (Invitrogen, Carlsbad, CA) using the following forward and reverse primers: PPAR-γ (NM_011146.2) forward primer 5'-TTT-TCA-AGG-GTG-CCA-GTT-TC-3' and reverse primer 5'-AAT-CCT-TGG-CCC-TCT-GAG-AT-3' (PCR product 197 bp); SREBP-1c (NM_011480.2) forward primer 5'-TGT-TGG-CAT-CCT-GCT-ATC-TG-3' and reverse primer 5'-AGG-GAA-AGC-TTT-GGG-GTC-TA-3' (PCR product 189 bp); C/EBP-α (NM_007678) forward primer 5'-TTA-CAA-CAG-GCC-AGG-TTT-CC-3' and reverse primer 5'-GGC-TGG-CGA-CAT-ACA-GTA-CA-3' (PCR product 189 bp); FABP4 (NM_024406) forward primer 5'-TCA-CCT-GGA-AGA-CAG-CTC-CT-3' and reverse primer 5'-AAT-CCC-CAT-TTA-CGC-TGA-TG-3' (PCR product 162 bp); FATP1 (NM_011977) forward primer 5'-TGC-CTC-TGC-CTT-GAT-CTT-TT-3' and reverse primer 5'-GGA-ACC-GTG-GAT-GAA-CCT-AA-3' (PCR product 161 bp); FAS (NM_007988.3) forward primer 5'-TTG-CTG-GCA-CTA-CAG-AAT-GC-3' and reverse primer 5'-AAC-AGC-CTC-AGA-GCG-ACA-AT-3' (PCR product 192 bp); ACS1 (NM_007981.3) forward primer 5'-CAA-CCC-AGA-ACC-ATG-GAA-GT-3' and reverse primer 5'-CTG-ACT-GCA-TGG-AGA-GGT-CA-3' (PCR product 195 bp); perillipin (NM_175640.1) forward primer 5'-AAG-GAT-CCT-GCA-CCT-CAC-AC-3' and reverse primer 5'-CCT-CTG-CTG-AAG-GGT-TAT-CG-3' (PCR product 191 bp); LPL (NM_008509.2) forward primer 5'-TCC-AAG-GAA-GCC-TTT-GAG-AA-3' and reverse primer 5'-CCA-TCC-TCA-GTC-CCA-GAA-AA-3' (PCR product 186 bp); β-actin (NM_007393.2) forward primer 5'-AGC-CAT-GTA-CGT-AGC-CAT-CC-3' and reverse 5'-TCC-CTC-TCA-GCT-GTG-GTG-GTG-AA-3' (PCR product 211 bp). PCR reactions were performed for 30 cycles, unless otherwise indicated, to identify the linear range for PCR. PCR products were separated on a 1.5% agarose gel and photographed. The PCR products were quantified using Flourchem software (Alpha Innotech, San Leandro, CA).

### Glucose uptake

Glucose uptake was analyzed by measuring the uptake of 2-deoxy-D-[^3^H] glucose as described previously [45,46]. Briefly, confluent 3T3-L1 adipocytes grown in 12 well plates were rinsed twice with serum free no-glucose DMEM medium, and incubated with 2 ml of the same medium in incubator with 8% CO_2 _at 37°C for 2 hours. The cells were washed with Krebs-Ringer-Hepes (KRP) buffer and incubated in 0.9 ml of this buffer at 37°C for 30 minutes. Insulin (0-200 nM) and RM (0-200 μg/ml) were added in the last 20 minutes. Glucose uptake was initiated by adding 0.1 ml of KRP buffer, 5 μCi/ml of 2-deoxy-D-[^3^H]-glucose and 1 mM of deoxy-glucose (final concentration). After 10 min, the glucose uptake was terminated by washing the cells thrice with cold PBS. The cells were lysed with 0.7 ml of 1% Triton X-100 at 37°C for 20 minutes, and the radioactive glucose uptake was determined by the scintillation counter (Beckman Inst. Inc, Fullerton, CA, USA).

### Western Blot analysis

3T3-L1 pre-adipocytes were treated with RM (0-300 μg/ml) during the process of differentiation as indicated in the text (perilipin analysis) and whole cell extracts prepared as previously described [47]. Alternatively, differentiated adipocytes were incubated in serum free media for 2 hours and subsequently stimulated with RM (0-100 μg/ml) or Insulin (0-200 nM) for 30 minutes (AMPK analysis). Equal amounts of cellular proteins (30 μg/lane) were separated on NuPAGE 12% or 4-12% Bis-Tris gel (Invitrogen, Carlsbad, CA, USA), and transferred to a PVDF membrane (Millipore, Bedford, MA, USA) using XCell II blot module (Invitrogen). The protein expression was detected with the corresponding primary antibodies for anti-perilipin, anti-phospho-AMPKα (Thr-172), anti-AMPK (Cell Signaling, Beverly, MA, USA) and anti-β-actin antibody (Santa Cruz Biotechnology, Santa Cruz, CA, USA). Protein expression was visualized using the ECL Western Blotting Detection System (Santa Cruz Biotechnology). Densitometric analysis of the bands for the expression of protein was done with Flourchem software (Alpha Innotech, San Leandro, CA).

### Statistical Analysis

Data are represented as mean ± S.D and were analyzed using SigmaPlot 11.2 (Systat Software Inc, San Jose, CA, USA). The differences between the groups were analyzed by one way ANOVA with Bonferroni multiple comparison post-hoc test.

## Results

### Effect of RM on lipid accumulation, triglyceride uptake and glycerol accumulation

**Figure 1:**
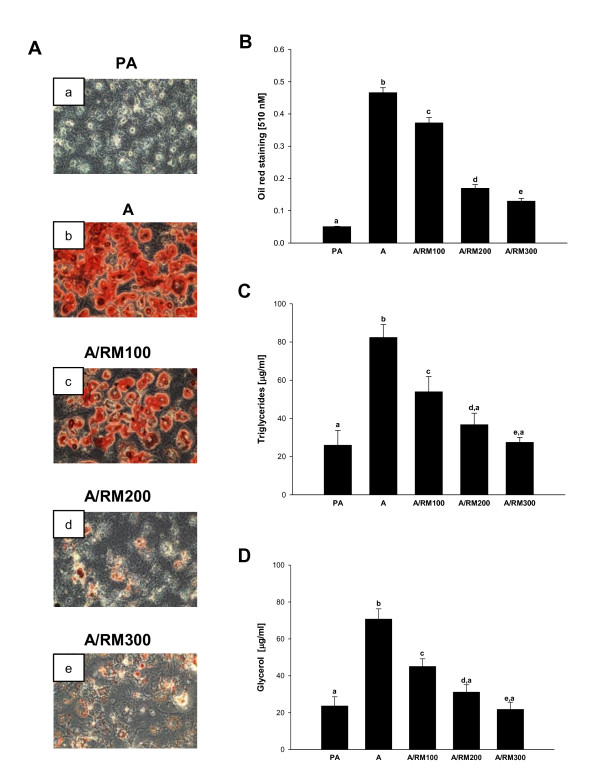
**Effect of RM on lipid accumulation during differentiation**. 3T3-L1 pre-adipocytes 2 days after confluence were induced to differentiate in the presence of RM (0-300 μg/ml). At day 9 adipocytes were stained with Oil red O as shown in Figure 1A. (a) pre-adipocytes, (b) adipocytes [RM-0], (c) adipocytes [RM-100 μg/ml], (d) adipocytes [RM-200 μg/ml], (e) adipocytes [RM-300 μg/ml]. (B) Quantification of lipids was measured after eluting with isopropanol and reading the absorbance at 510 nm. (C) triglyceride and (D) glycerol concentration was determined as described in *Materials and Methods*. Each experiment was performed three times in triplicates. Results are depicted as mean ± SD, statistical significance was calculated (letters that differ indicate, p ≤ 0.05). For our studies we used cells up to passage twelve.

To evaluate whether RM affects the lipid uptake in adipocytes, we analyzed the changes in the amount of triglycerides and glycerol after inducing adipocyte differentiation. Adipocytes accumulate lipids by two major mechanisms either by *de novo *lipogenesis from non-lipid precursors or by uptake of fatty acids from the plasma [48]. To evaluate the effect of RM on lipid uptake by adipocytes, cells were treated with RM (0-300 μg/ml) in the presence of the differentiation mix of 1 μg/ml of INS, 0.25 μM of DEX and 0.5 mM IBMX in DMEM with 10% FBS as described in *Materials and Methods*. As seen in Figure 1A, differentiation of preadipocytes (a) to adipocytes (b) was associated with the increased oil red O positive cells, whereas RM treatment markedly suppressed lipid accumulation in differentiated adipocytes (Figure 1A b-e, Figure 1B). Since most reserves of energy in the human body are stored in adipocytes in the form of triacylglycerol (TAG) [12], we assessed the amount of triglycerides (TG) and glycerol released due to lipolysis from adipocytes treated with RM. As expected, TG and glycerol levels in adipocytes were significantly decreased in a dose response manner after RM treatment (Figure 1C and Figure 1D).

### RM inhibits the expression of transcription factors controlling lipogenic pathways

**Figure 2:**
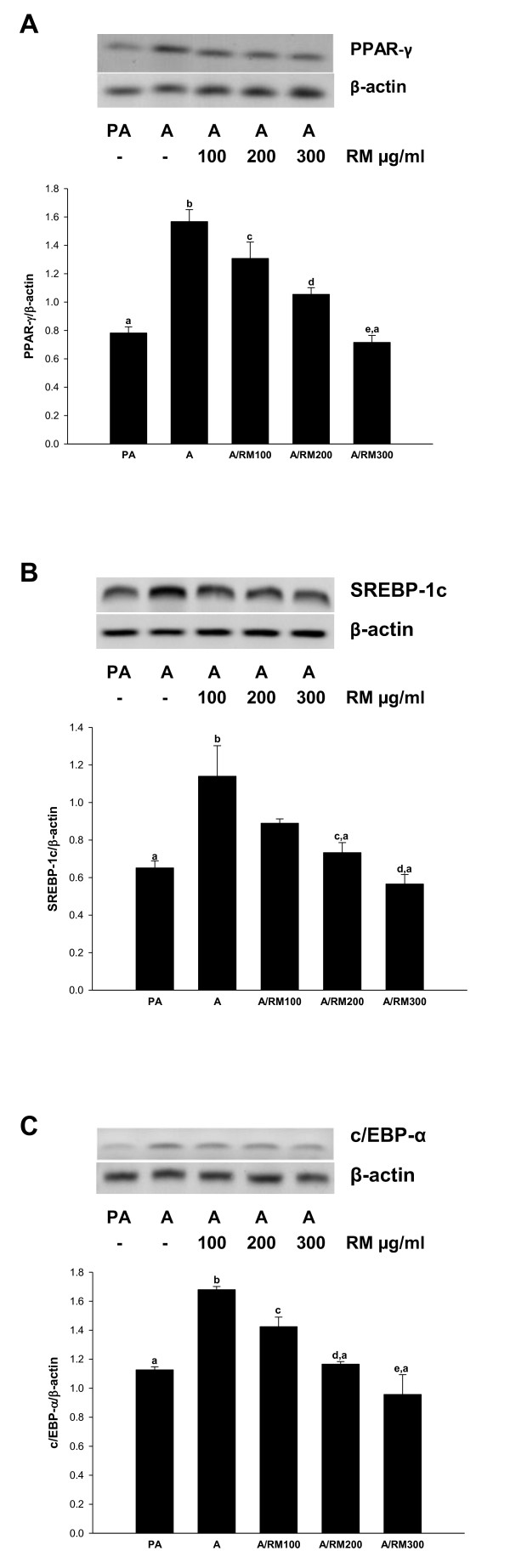
**Effect of RM on adipogenic transcription factors**. 3T3-L1 pre-adipocytes 2 days after confluence were induced to differentiate in the presence of RM (0-300 μg/ml). At day 9 adipocytes RNA was isolated and RT-PCR for mRNA expression of (A) PPAR-γ, (B) SREBP-1 and (C) c/EBP-α was performed as described in *Materials and Methods*. Ratio is calculated against β-actin represented as arbitary units of integrated density value (IDV). Each experiment was repeated twice in duplicate and results are depicted as mean ± SD, statistical significance was calculated (letters that differ indicate, p ≤ 0.05).

Next we examined the role of transcription factors involved in adipocyte differentiation. The effect of RM on the expression of PPAR-γ, SREBP-1c and C/EBP-α was evaluated by RT-PCR. As seen in Figure 2, differentiated adipocytes had substantially increased levels of the PPAR-γ, SREBP-1c and C/EBP-α compared to pre-adipocytes. Moreover, treatment of pre-adipocytes with ReishiMax (0-300 ug/ml) decreased the expression of PPAR-γ (Figure 2A), SREBP-1c (Figure 2B) and C/EBP-α (Figure 2C) in a dose dependent manner, suggesting that the RM inhibits adipocyte lipogenesis through PPAR-γ, SREBP-1c and C/EBP-α regulated genes.

### RM suppresses expression of proteins regulating synthesis and transport of fatty acids

**Figure 3:**
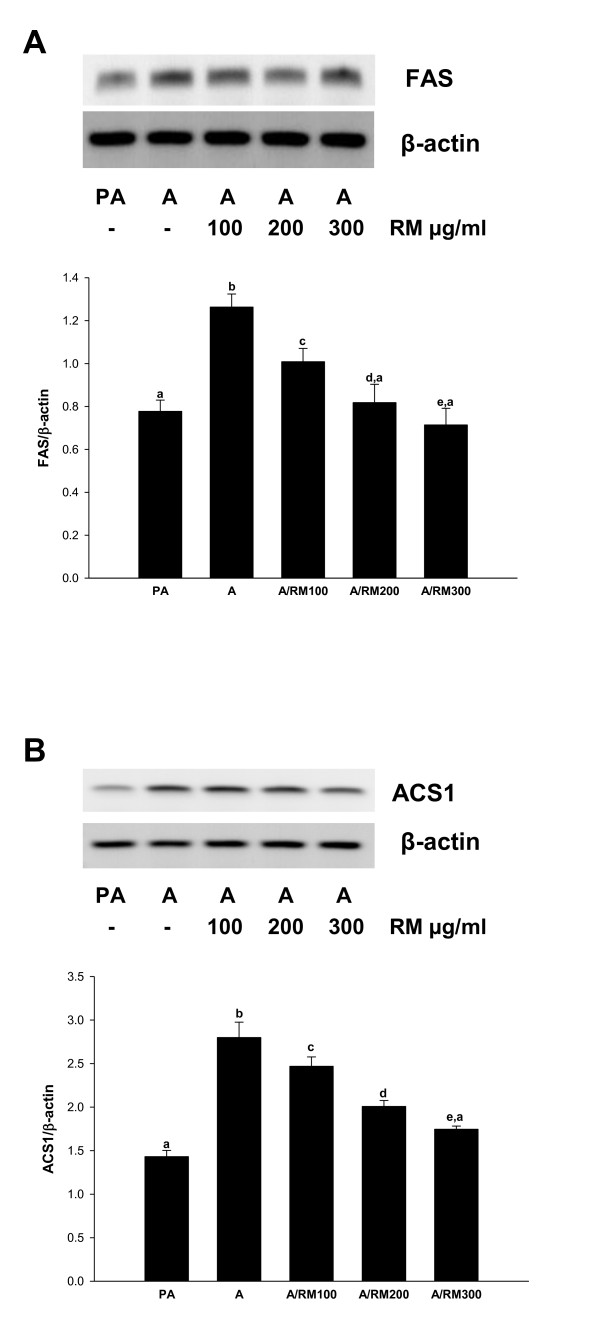
**Effect of RM on FAS and ACS1**. 3T3-L1 pre-adipocytes 2 days after confluence were induced to differentiate in the presence of RM (0-300 μg/ml). At day 9 adipocytes RNA was isolated and RT-PCR for mRNA expression of (A) fatty acid synthase (FAS) and (B) acyl-coA synthetase (ACS1) was performed. Ratio is calculated against β-actin represented as arbitary units of integrated density value (IDV). Each experiment was repeated twice in duplicate and results are depicted as mean ± SD, statistical significance was calculated (letters that differ indicate, p ≤ 0.05).

**Figure 4:**
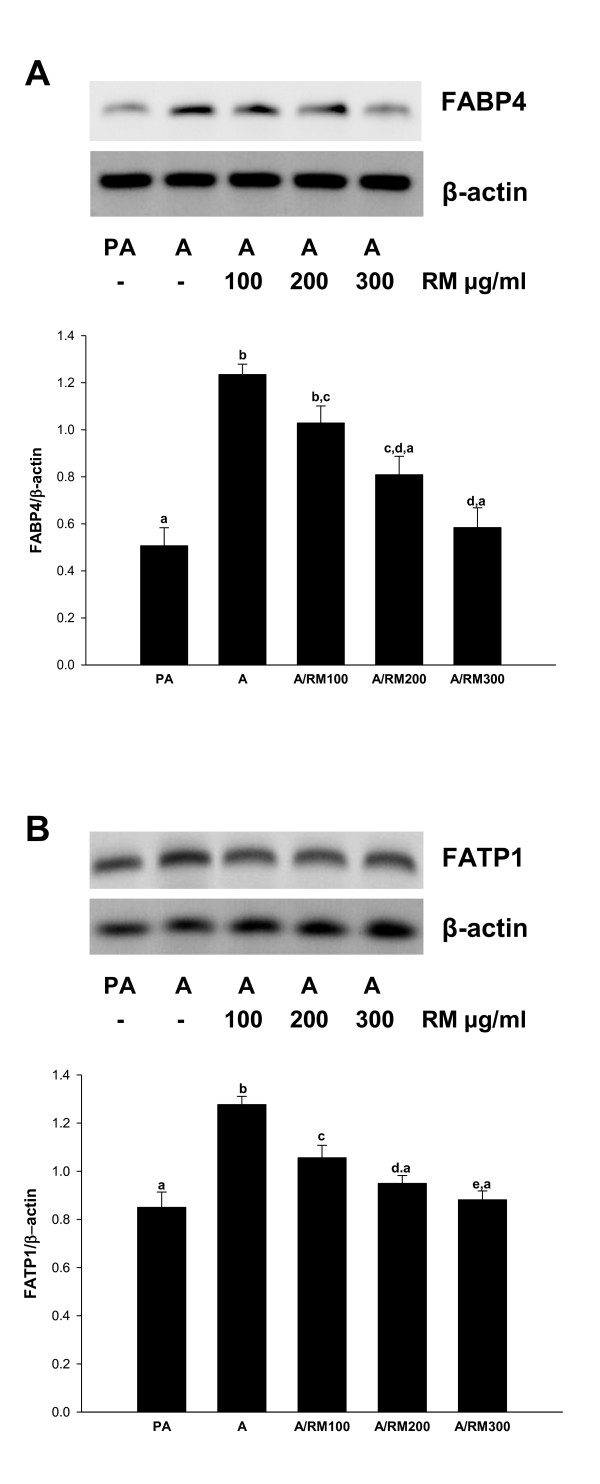
**Effect of RM on FABP4 and FATP1**. 3T3-L1 pre-adipocytes 2 days after confluence were induced to differentiate in the presence of RM (0-300 μg/ml). At day 9 adipocytes RNA was isolated and RT-PCR for mRNA expression of (A) fatty acid binding protein 4 (FABP4) and (B) fatty acid transport protein 1 (FATP1) was performed. Ratio is calculated against β-actin represented as arbitary units of integrated density value (IDV). Each experiment was repeated twice in duplicate and results are depicted as mean ± SD, statistical significance was calculated (letters that differ indicate, p ≤ 0.05).

As shown above, RM treatment inhibits the expression of PPAR-γ, SREBP-1c and C/EBP-α which regulate genes responsible for the synthesis and transport of fatty acids. Thus, we determined whether RM also suppresses expression of the fatty acid synthesis enzymes (FAS and ACS1) and proteins involved in the transport of fatty acids (FABP4 and FATP1). 3T3-L1 cells were differentiated and treated with RM (0-300 ug/ml) and expression of FAS, ACS1, FABP4 and FATP1 evaluated by RT-PCR as described in *Materials and Methods*. As seen in Figure 3A and 3B, RM treatment significantly inhibited the expression of FAS and ACS1 in a dose-response manner. In addition, RM treatment significantly inhibited the expression of FABP4 (Figure 4A), and FATP1 (Figure 4B), Therefore our data suggest that RM suppresses expression of proteins involved in lipid synthesis and lipid transport.

### Effect of RM on the expression of perilipin and LPL

**Figure 5:**
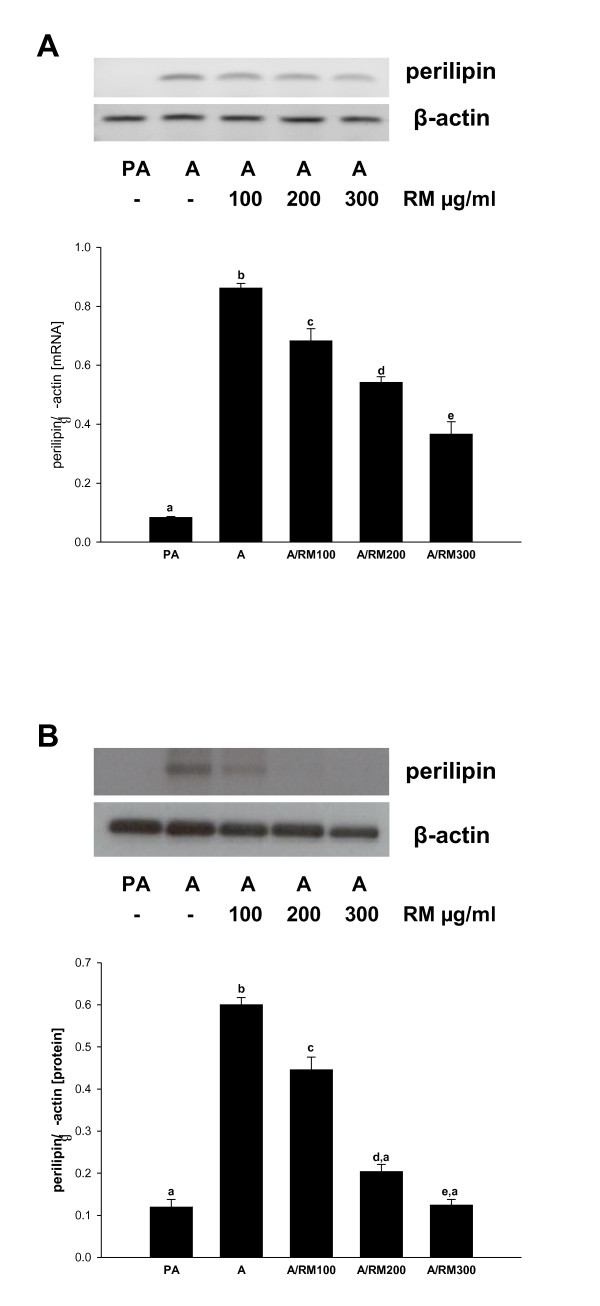
**Effect of RM on mRNA levels of perilipin and protein expression for perilipin**. 3T3-L1 pre-adipocytes 2 days after confluence were induced to differentiate in the presence of RM (0-300 μg/ml). At day 9 adipocytes RNA and proteins were isolated. (A) mRNA levels of perilipin were determined by RT-PCR and (B) protein expression of perilipin was determined by Western blot analysis. Each experiment was repeated thrice and results are depicted as mean ± SD, statistical significance was calculated (letters that differ indicate, p ≤ 0.05).

During the process of differentiation the adipocytes accumulated lipid in the form of lipid droplets. These lipid droplets are protected by different fat specific proteins including perilipin. Since RM treatment decreases the expression of the transcription factors, enzymes and transporter proteins involved in the synthesis and transport of fatty acids, we were interested to further determine whether RM treatment could also modulate the expression of perilipin. As seen in Figure 5, differentiation from preadipocytes to adipocytes markedly increased the levels of perilipin, whereas RM suppressed dose-dependently its expression at mRNA (Figure 5A) as well as protein (Figure 5B) levels, respectively. Lipid droplets are hydrolyzed by lipoprotein lipase (LPL) [49] which expression is controlled by SREBP-1c during adipocyte differentiation [50]. Since RM inhibits production of glycerol and SREBP-1c (Figure 1D, 2B), we evaluated the effect of RM (0-300 ug/ml) on LPL expression by RT-PCR during the adipocyte differentiation. However, RM did not show any effect on LPL expression (not shown).

### RM induces AMPK and glucose uptake in adipocytes

**Figure 6:**
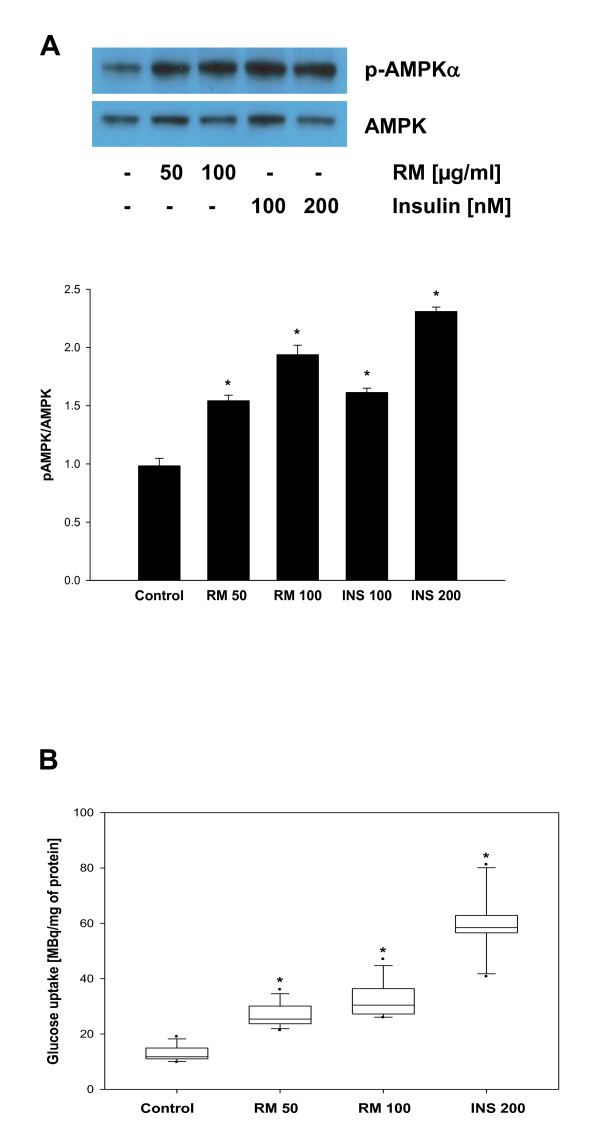
**Effect of RM on AMPK and glucose uptake**. 3T3-L1 pre-adipocytes 2 days after confluence were induced to differentiate. (A) 3T3-L1 adipocytes were treated with RM (0, 50, 100 μg/ml) or insulin (100, 200 nM) for 30 minutes and the expression of phopho-AMPK was detected by Western blot analysis as described in *Materials and Methods*. Immunoblots are representative of 3 independent experiments. Data represents mean ± SD. *p < 0.05 vs adipocyte control cells. (B) Glucose uptake was determined in 3T3-L1 adipocytes treated with RM (0, 50, 100 μg/ml) or insulin (200 nM) as described in *Materials and Methods*. The results are mean ± SD (n = 12), *p < 0.05 vs adipocyte control cells.

The activation/phosphorylation of AMPK is crucial for the treatment of diabetes and metabolic syndrome [51]. To evaluate whether RM activates AMPK, 3T3-L1 adipocytes were treated with RM (0, 50, 100 μg/ml) or insulin (100, 200 nM) for 30 minutes and phosphorylation of AMPK determined by Western blot analysis with anti-pAMPK-Thr172 antibody. As seen in Figure 6A, RM induced phosphorylation of AMPK in a dose response manner and this effect was comparable to the insulin treatment. Because AMPK can function as an energy sensor and also stimulates glucose uptake [52,53], we evaluated whether RM stimulates glucose uptake in differentiated adipocytes. As expected, RM markedly induced glucose uptake in adipocytes in a dose response manner (Figure 6B), confirming that RM-induced phosphorylation of AMPK results in the glucose uptake.

## Discussion

Obesity is increasing at an alarming rate in both the genders of all age groups around the world including United States [54]. According to the National Bureau of Economic Research report, the prevalence of obesity will rise to 40% in men and 43% in women by 2020 [55]. The major factors contributing to obesity are sedentary life style, inactivity, over eating and disturbances in the metabolism of fatty acids [56]. Various studies indicated that obese humans are at increased risk of developing diabetes, hypertension, cancer and atherosclerosis [57-59]. Thus, adipose tissue is recognized as a major secretory organ, releasing a variety of adipocytokines (e.g. adiponectin, leptin, resitin and others) which provide the link between obesity, insulin resistance and inflammatory disorders [60]. Therefore, new therapeutic agents that can regulate adipogenesis and glucose uptake can be employed to control obesity and type 2 diabetes.

**Figure 7:**
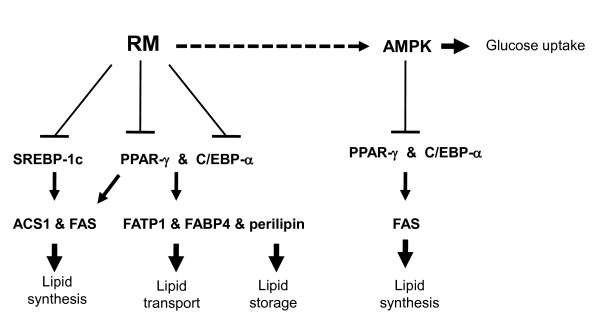
**Schematic of RM activity**.

In the present study, we evaluated the effect of ReishiMax (RM), dietary supplement containing triterpenes and polysaccharides isolated from medicinal mushroom *Ganoderma lucidum*, on adipocyte differentiation and glucose uptake in adipocytes. Here, we show that RM inhibited adipocyte differentiation/lipid accumulation through: a) the down-regulation of expression of transcription factors PPAR-γ, SREBP-1c and C/EBP-α, and b) the suppression of expression of genes responsible for lipid synthesis (FAS, ACS1), lipid transport (FABP4, FATP1) and lipid storage (perillipin). However, we did not observe any changes in the expression of LPL, which expression is also controlled by PPAR-γ, SREBP-1c and C/EBP-α. In agreement with our study, hydroxytorosol from olive oil inhibited lipid accumulation during adipocyte differentiation and inhibited all tested genes except LPL [61]. Nevertheless, others demonstrated down-regulation of LPL expression associated with the adipocyte differentiation [62,63]. These contradictory results might be explained by the use of different cell models: mouse 3T3-L1 (our study and [63]), C3H10 T1/2 [61] or human pre-adipocytes [62] or activation of β-catenin pathway [63]. Finally, RM activated AMPK resulting in the increased glucose uptake by adipocytes (Figure 7).

On the molecular level, C/EBP-α, PPAR-γ and SREBP-1c are induced during adipocyte differentiation [12]. Specifically, PPAR-γ and SREBP-1c mRNAs start to be expressed at the very early/early stage (day 1-2 post-confluence) followed by the expression of C/EBP-α at the intermediate stage (day 4 post-confluence) [50]. These transcription factors further controls expression of adipocyte-specific genes (e.g. FAS, ACS1, FABP4, FATP1 and perilipin) at the late stage (day 5 post-confluence) leading to the fat droplet formation [64]. Therefore, the inhibition of PPAR-γ, SREBP-1c and C/EBP-α, by RM at the early/intermediate stages results in the suppression of expression of adipocyte specific genes and lipid formation at the late stage of adipocyte differentiation.

The inhibition of adipogenesis by RM is in an agreement with recent studies demonstrating anti-adipogenic effects of white tea or bitter melon extracts or other isolated phytochemicals [65-69]. Moreover, activation of AMPK was previously associated with the inhibition of adipocyte differentiation [70] or increased glucose uptake. Indeed, our data are in an agreement with recent reports by Ha *et al *[71,72] demonstrating an increase of glucose uptake and inhibition of adipocyte differentiation through the activation of AMPK in 3T3-L1 cells.

During the preparation of the present manuscript, Lee *et al*. demonstrated that some purified triterpenes from *G. lucidum *inhibit adipocyte differentiation, decrease lipid accumulation and down-regulate expression of PPAR-γ, SREBP-1c, C/EBP-α, FAS and ACC [73-75]. Although the identification of biologically active components from *G. lucidum *is important for their possible therapeutic use, and more than 130 triterpenes have been isolated and new triterpenes continue to be identified [76], the yield of a majority of purified triterpenes is generally low. Therefore, the possible therapeutic use of purified triterpenes is currently limited. On the other hand, in our study we used standardized dietary supplement ReishiMax (RM) containing 6% triterpenes and 13.5% polysaccharides. Moreover, we have previously identified some of the triterpenes in RM (e.g. ganoderic acids A, F, H, Mh, S, lucidenic acid B, D, D1, E1, L and methyl lucidenate G) [77], that can be used for the standardization of the active supplements or *G. lucidum *extracts. Our study is with agreement with previous study demonstrating stimulation of glucose uptake and activation of AMPK in rat muscle cells [78]. However, the authors used uncharacterized extract prepared from *G. lucidum *purchased from the Korean market [78]. On the other hand *G. lucidum *extract (GE), containing approximately 10% of ganoderic acid A, induced adipocyte differentiation and expression PPAR-γ [79]. Although GE activated PPAR-γ, ganoderic acid A itself did not show any effect on PPAR-γ, suggesting that other compounds in GE are responsible for the GE activity [79]. Finally, standardized *G. lucidum *extract, containing polysaccharides, adenosine and ganoderic acid A, demonstrated mild anti-diabetic effects in a controlled human trial [80]. Since the presence and the amount of specific biologically active compounds in *G. lucidum *extracts depends on the source of *G. lucidum*, cultivation conditions, storage and extraction process [81], it is not surprising that extracts from the same mushroom could have different or opposite activities.

In summary, only standardized and characterized dietary supplements/extracts should be considered for the use in alternative medicine. Since the use of dietary supplements is not regulated and they are freely accessible, the proper characterization must be performed and associated with the specific activity.

## Conclusions

In conclusion, the results of our study show that chemically characterized dietary supplement ReishiMax [RM], containing triterpenes and polysaccharides isolated from medicinal mushroom *Ganoderma lucidum*, inhibits the expression of key transcription factors and genes responsible for adipocyte differentiation, synthesis, transport and storage of lipids. In addition, RM activates AMPK and stimulates glucose uptake to the levels comparable to the insulin activity. Further preclinical studies evaluating RM activity in the management of obesity and type 2 diabetes are warranted.

## List of abbreviations

ACS1: acyl-CoA synthetase-1; AMPK: AMP-activated protein kinase; C/EBP-α: CCAAT/enhancer binding protein-α; FABP4: fatty acid binding protein-4; FAS: fatty acid synthase; FATP1: fatty acid transport protein-1; LPL: lipoprotein lipase; PPAR-γ: peroxisome proliferator-activated receptor-γ; RM: ReishiMax; SREBP-1c: sterol regulatory element binding element protein-1c.

## Competing interests

The authors declare that they have no competing interests.

## Authors' contributions

DS designed research; AT-S and BL conducted research; AT-S, BL and DS analyzed data; AT-S and DS wrote the paper; DS had the primary responsibility for final content. All authors read and approved the final manuscript.

## Pre-publication history

The pre-publication history for this paper can be accessed here:

http://www.biomedcentral.com/1472-6882/11/74/prepub
